# Complex evaluation of serum immunoglobulin levels in patients with chronic lymphocytic leukemia: Significant increase in IgA after first‐line chemoimmunotherapy

**DOI:** 10.1002/cam4.7399

**Published:** 2024-08-09

**Authors:** Pavel Vodárek, Dominika Écsiová, Vladimíra Řezáčová, Ondřej Souček, Martin Šimkovič, Doris Vokurková, David Belada, Pavel Žák, Lukáš Smolej

**Affiliations:** ^1^ 4th Department of Internal Medicine—Hematology University Hospital Hradec Kralove Hradec Kralove Czech Republic; ^2^ Faculty of Medicine in Hradec Kralove, Charles University Hradec Kralove Czech Republic; ^3^ Institute of Clinical Immunology and Allergology University Hospital Hradec Kralove Hradec Kralove Czech Republic

**Keywords:** chemoimmunotherapy, CLL, immunoglobulin, immunosuppression, infections, prognosis

## Abstract

**Introduction:**

The impact of chemoimmunotherapy (CIT) on immunoglobulin (Ig) quantities in patients with chronic lymphocytic leukemia (CLL) has not been extensively studied.

**Methods:**

We analyzed Ig levels in 45 stable patients with indolent CLL (without indication for treatment) and 87 patients with progressive disease before first‐line treatment. Fifty‐five patients were evaluated again after the treatment with CIT.

**Results:**

We observed significantly lower levels of all Ig classes and subclasses in patients with progressive disease compared to patients with indolent disease. After treatment, median IgA increased from 0.59 g/L to 0.74 g/L (*p* = 0.0031). In stable patients, lower IgA2 was associated with shorter time to first treatment, although it did not reach statistical significance (*p* = 0.056). Shorter overall survival was observed in patients with progressive disease and lower IgG2 (*p* = 0.043). Surprisingly, among the patients with progressive CLL, unmutated IGHV genes were associated with higher levels of IgG, IgG1 and IgM, while TP53 mutation and/or 17p deletion were associated with higher levels of IgA and IgA1.

**Conclusions:**

CIT may lead to increase in IgA levels. Hypogammaglobulinemia is more common in patients with progressive CLL and unmutated IGHV or TP53 dysfunction.

## 1 INTRODUCTION

In Europe and North America, chronic lymphocytic leukemia (CLL) has the highest prevalence of all the leukemias of the adults.^1^ One of its typical features is an extremely heterogeneous clinical course—while some patients die early after the diagnosis, others survive for many years without any need for treatment. Therefore, many prognostic markers, such as Rai or Binet stage, cytogenetic aberrations, mutational status of the immunoglobulin heavy chain variable region (IGHV), or mutation of the tumor protein 53 (*TP53*) gene, have been established to help identify patients with an early need for treatment.^2^ Another typical feature of CLL is a complex alteration of the immune system, resulting in progression of the disease, autoimmune complications, second malignancies, and a higher frequency of infections.^3^, ^4^ Infections are the most important cause of morbidity and mortality in CLL patients, affecting more than 80% throughout the disease course, and are the cause of death in up to 60%.^4^ The longest known immune defect in CLL patients is hypogammaglobulinemia, described since the 1960s.^5^ Its prognostic significance and connection with infectious complications have been investigated in a number of studies, sometimes with contradictory results.^6^, ^7^, ^8^, ^9^, ^10^, ^11^, ^12^, ^13^, ^14^, ^15^, ^16^, ^17^, ^18^, ^19^, ^20^ Typical pathogens associated with hypogammaglobulinemia are encapsulated bacteria commonly causing respiratory infections: *Staphylococcus aureus*, *Haemophilus influenzae*, or *Streptococcus pneumoniae*.^21^

Only some researchers have investigated the possible association between hypogammaglobulinemia and prognostic factors. Neither age, sex, β2 microglobulin level, CD38 or ZAP70 positivity, nor unfavorable cytogenetic aberrations or unmutated IGHV were associated with low immunoglobulin (Ig) levels in a study by Mauro et al.^6^ Others observed an association of hypogammaglobulinemia with advanced stage, CD49d positivity, or higher leukocyte count.^7^, ^8^

The mechanism leading to low Ig levels in CLL patients is complex. CLL cells can inhibit bone marrow plasma cells through the CD95 (Fas receptor on plasma cells)–CD95 L (Fas ligand on CLL cells) interaction.^22^ Additionally, inhibition of plasma cells by autologous NK cells has been described.^23^ In a study of Criado et al., a direct correlation was found between total Ig levels and the number of normal residual B cells among CLL patients.^24^ Various defects in T cells and the related inability to form specific immune responses probably contribute as well.^25^

The last decade has seen chemoimmunotherapy (CIT) being replaced with targeted treatment with the Bcl‐2 inhibitor venetoclax and Bruton tyrosine kinase inhibitors (BTKi). Nevertheless, some CIT regimens are still relatively widely used, especially where newer treatment options are less affordable.^26^ CIT is generally believed to further aggravate disease‐related immunosuppression, including hypogammaglobulinemia, but there is still insufficient information specifically about currently used CIT regimens and their effect on Ig levels in patients with CLL.^27^, ^28^, ^29^, ^30^, ^31^, ^32^, ^33^ In contrast, ibrutinib treatment was repeatedly shown to increase IgA levels, while mostly having no effect on IgG and IgM.^34^, ^35^, ^36^, ^37^, ^38^

### 1.1 Aims of the study

To assess differences in Ig levels between patients with indolent disease (without indication for treatment) and patients with progressive disease before first‐line treatment. In addition, changes after first‐line treatment with CIT were evaluated, as well as associations between the Ig levels, disease course, known prognostic factors, and frequency of infections.

## 2 METHODS

### 2.1 Patients and data acquisition

All patients were diagnosed with CLL according to the 2008 International Workshop on CLL (IWCLL) criteria.^39^ All of them also signed informed consent forms, the study was approved by the local ethics committee and conducted according to the principles of the Declaration of Helsinki. The study had single‐center design—all patients were prospectively followed at the 4th Department of Internal Medicine – Hematology, University Hospital in Hradec Králové, Czech Republic, from September 2013 to November 2020.

Samples of peripheral blood were collected from patients immediately before the treatment and 2–3 months after treatment completion. As for the patients with indolent disease course who were not treated, their samples were collected just upon entry into the study. None of the patients received any form of Ig replacement. The treatment indication was based on the IWCLL criteria.^39^ CIT regimens used were as follows: fludarabine + cyclophosphamide + rituximab (FCR), bendamustine + rituximab (BR), obinutuzumab + chlorambucil (O‐Clb) and rituximab + chlorambucil (R‐Clb). The dosing was standard and corresponded to those used in CLL10, CLL11 a CLL 208 studies.^40^, ^41^, ^42^ Information on known prognostic factors (Rai stage, fluorescence in situ hybridization (FISH) and karyotyping results, IGHV and *TP53* mutational status), as well as information on frequency, severity, and site of infections were collected from patients’ records. TP53 status was investigated by Sanger sequencing, IGHV mutational status was obtained after amplification of IGHV gene transcript by polymerase chain reaction with subsequent sequencing of product with Sanger method and comparing the result to germline genes in Ig databases. Time to first treatment (TTFT), time to next treatment (TTNT), and overall survival (OS) were calculated from the time of sample collection. Control group was established from healthy blood donors.

Serum levels of IgG, IgG subclasses IgG1–4, IgA, IgA subclasses IgA1–2, and IgM were measured by immunonephelometry using the immunochemistry system Image by Beckman Coulter (Miami, FL, USA). All patients also underwent serum protein electrophoresis, and if a narrow spike in the gamma zone was detected, immunofixation electrophoresis was added to detect a potential paraprotein. Measurements of patients with paraproteins were not included in the statistical analysis for a particular Ig class.

### 2.2 Statistics

Different cohorts of patients were compared by use of the Mann–Whitney *U*‐test, while paired samples of the same patients before and after the treatment were compared using the Wilcoxon test. Correlation analysis was used to assess relationship between Ig levels and age or absolute lymphocyte count (ALC). Association between Ig levels and OS, TTFT, or TTNT was explored with the Cox proportional hazards regression method. The Kaplan–Meier method was used for survival analysis. The ideal cut‐off values for Ig quantities to allow for the best separation of the survival curves were determined by receiver operating characteristic (ROC) curve. Statistical significance was defined as *p* value ≤0.05 Statistical analysis was performed with MedCalc software, version 20 (MedCalc Software Ltd, Mariakerke, Belgium).

## 3 RESULTS

**FIGURE 1:**
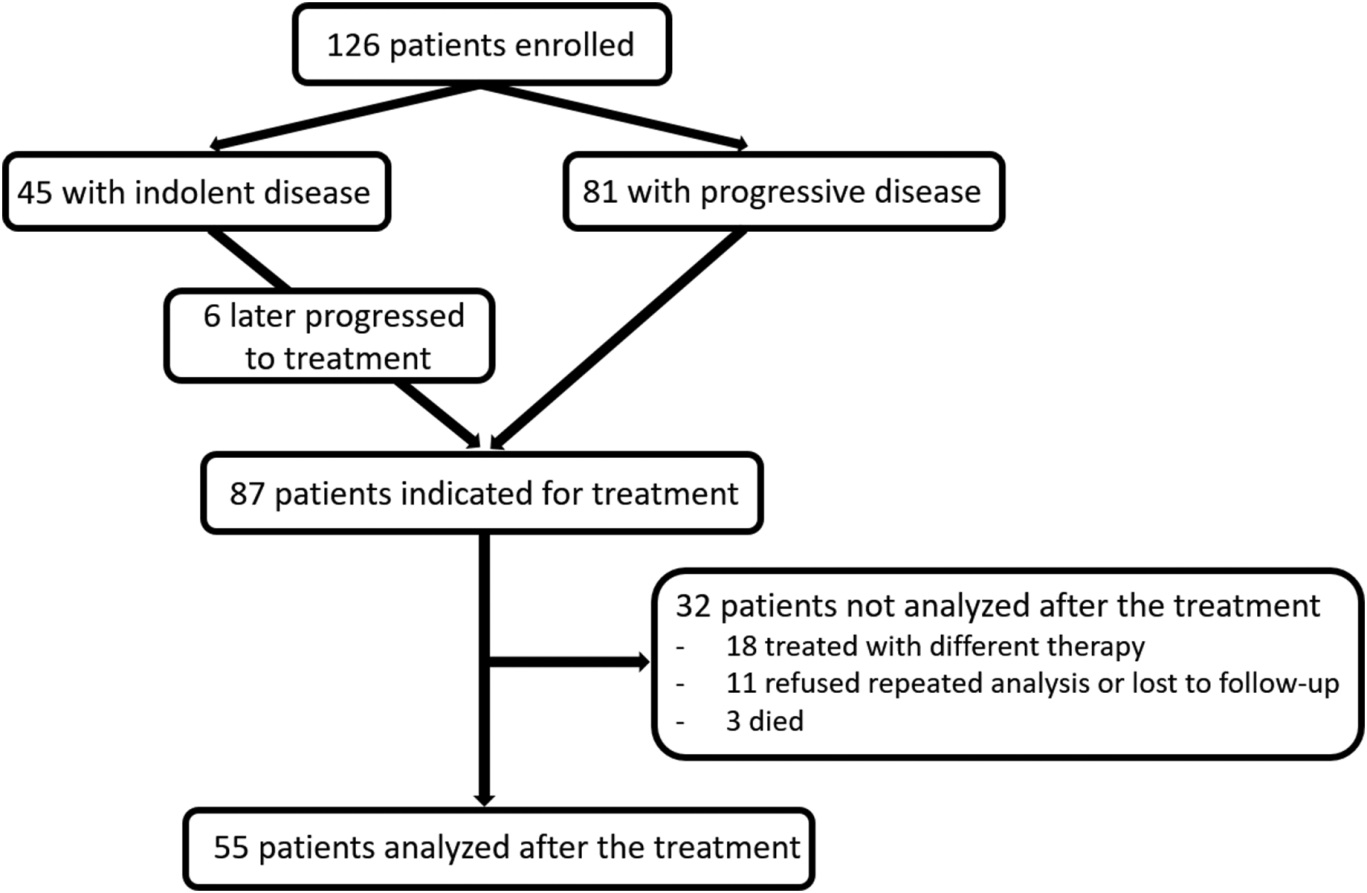
Flow chart describing the patients' disposition.

Patients were enrolled into the study from September 2013 to May 2019, and then followed up until November 2020. Out of 126 enrolled patients, 45 were without indication for treatment (group with indolent, stable disease), while 81 had progressive CLL indicated for treatment. Six patients were analyzed as part of the stable cohort and later again, once their disease progressed. These six were not used for comparison between stable and progressive cohort, but they were included in comparison of Ig values before and after CIT. Blood sample after therapy was taken in 55 patients. The rest of them were treated with regimens other than R‐Clb, O‐Clb, BR or FCR (usually high‐dose corticosteroids or targeted treatment) or their blood samples were lost or not taken for various reasons. The control group consisted of 45 healthy individuals (median age 61 years [range, 52–73], 44% males). Basic characteristics of all other cohorts are shown in Table 1 and flow chart describing the patients’ disposition is provided in Figure 1. Partial or complete remission was achieved in 8/11 patients after R‐Clb, 8/9 after O‐Clb, 15/17 after BR and in all 18 patients after FCR.

**TABLE 1:** Characteristics of the patient cohorts.

Characteristic	Stable	Progressive	After the treatment
Total number	45	87	55
Age, median (range), years at the date of sample collection	65 (34–88)	69 (43–87)	68 (43–87)
Males	24 (53%)	56 (64%)	31 (56%)
Median follow‐up from the date of blood collection	49 months	26 months	30 months
Median time (range) from diagnosis to the date of blood collection	26 months (4–141)	33 months (0–174)	42 months (1–174)
Rai modified risk at the date of blood collection			
Low	27 (60%)	1 (1%)	1 (2%)
Intermediate	16 (36%)	34 (39%)	22 (40%)
High	2 (4%)	52 (60%)	32 (58%)
IGHV			
Mutated	25 (56%)	19 (22%)	13 (24%)
Unmutated	10 (22%)	55 (63%)	33 (60%)
NA	10 (22%)	13 (15%)	9 (16%)
*TP53*			
Mutated	0	9 (10%)	5 (9%)
Unmutated	30 (67%)	64 (74%)	39 (71%)
NA	15 (33%)	14 (16%)	11 (20%)
FISH			
Normal	9 (20%)	16 (18%)	13 (24%)
13q deletion	24 (53%)	26 (30%)	18 (33%)
12 trisomy	4 (9%)	16 (18%)	9 (16%)
11q deletion	2 (4%)	15 (17%)	8 (15%)
17p deletion	0	9 (10%)	2 (4%)
NA	6 (13%)	5 (6%)	5 (9%)
*TP53* mutation or 17p deletion	0	13 (15%)	6 (11%)

*Note*: Data for the “After the treatment” cohort correspond to the pretreatment values. Rai modified risk: stage 0 = low, stage I/II = intermediate, stage III–IV = high.

Abbreviations: FISH, cytogenetic aberrations detected by fluorescence in situ hybridization; IGHV, mutational status of the immunoglobulin heavy chain variable region; NA, not available. *TP53*, mutation of tumor protein 53 investigated by Sanger sequencing.

### 3.1 Comparison among controls, patients with stable and progressive disease and the effect of treatment

In the stable patient cohort (*n* = 45), one patient had IgG paraprotein. In patients with progressive disease (*n* = 87), paraproteins in the IgG/IgA/IgM class were detected in 7/1/5 patients. In patients analyzed before and after treatment (*n* = 55), paraproteins in the IgG/IgA/IgM class were detected in 3/0/3 patients. All patients with detected paraproteins were excluded from further analysis for a particular Ig class and its subclasses. All three patients’ cohorts (stable, progressive and patients after treatment) had significantly lower levels of all Ig classes and subclasses than controls, with single exception of IgG3 (detailed in Tables S1–S3). The number of patients with hypogammaglobulinemia in each class and subclass is described in Table 2.

**TABLE 2:** Number of patients with hypogammaglobulinemia in each class and subclass.

Immunoglobulin class (lower limit of normal)	Number of patients with hypogammaglobulinemia within each cohort			
	Stable	Progressive	Before the treatment	After the treatment
IgG (7.3 g/L)	11 (25%)	44 (55%)	29 (56%)	29 (56%)
IgA (0.8 g/L)	10 (22%)	55 (63%)	36 (65%)	29 (53%)
IgM (0.4 g/L)	13 (29%)	45 (55%)	27 (52%)	30 (58%)
IgG1 (3.6 g/L)	7 (16%)	26 (33%)	18 (36%)	19 (37%)
IgG2 (1.55 g/L)	3 (7%)	26 (26%)	17 (33%)	11 (21%)
IgG3 (0.19 g/L)	4 (9%)	17 (21%)	8 (15%)	6 (12%)
IgG4 (0.08 g/L)	3 (7%)	8 (10%)	4 (8%)	8 (15%)
IgA1 (0.67 g/L)	8 (18%)	51 (59%)	32 (58%)	29 (53%)
IgA2 (0.06 g/L)	2 (4%)	12 (14%)	9 (16%)	2 (4%)

**FIGURE 2:**
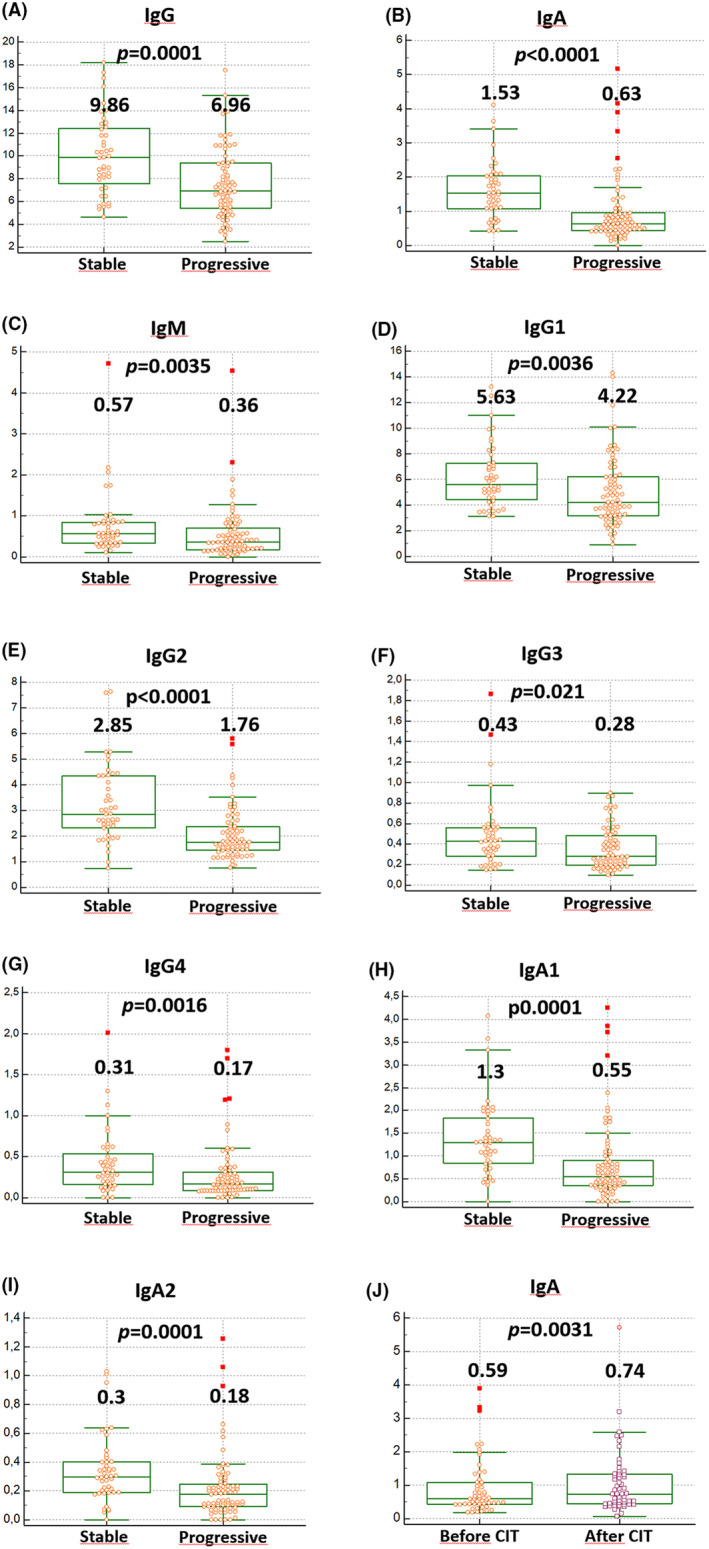
Box and whisker plots comparing immunoglobulin levels in patients with stable and progressive disease (A–I) and IgA of patients before and after treatment (J). Quantities of immunoglobulins are expressed as medians in g/L. The boxes represent the interquartile range, and the whiskers are drawn at a distance of 1.5 times the interquartile range from the first and third quartiles.

With the exception of IgG3, we observed significantly lower levels of all Ig classes and subclasses in patients with progressive disease (*n* = 81) compared to patients with stable disease (*n* = 45) (Table 3 and Figure 2). Regarding paired samples before versus after therapy (*n* = 55), the only significant change was an increase in IgA (Table 4 and Figure 2). As Ig levels did not change significantly after treatment (except for IgA), they remained significantly lower than those of stable patients (Table 5).

**TABLE 3:** Comparison of patients with stable and progressive disease.

Immunoglobulin class	Stable	Progressive	*p* value
IgG	9.86	6.96	0.0001
IgA	1.53	0.63	<0.0001
IgM	0.57	0.36	0.0035
IgG1	5.63	4.22	0.0036
IgG2	2.85	1.76	<0.0001
IgG3	0.43	0.28	0.021
IgG4	0.31	0.17	0.0016
IgA1	1.3	0.55	<0.0001
IgA2	0.3	0.18	0.0001

*Note*: Quantities of immunoglobulins are expressed as medians in g/L.

**TABLE 4:** Comparison of patients before and after treatment (paired samples).

Immunoglobulin class	Before treatment	After treatment	Median/Mean of fold‐changes	*p* value
IgG	7.12	6.83	1.08/1.1	NS
IgA	0.59	0.74	1.13/1.28	0.0031
IgM	0.39	0.36	0.97/1.17	NS
IgG1	4.09	4.2	1.06/1.13	NS
IgG2	1.72	1.97	1.11/1.18	NS
IgG3	0.3	0.39	1.12/1.36	NS
IgG4	0.18	0.16	0.98/1.22	NS
IgA1	0.54	0.65	1.04/1.25	NS
IgA2	0.13	0.19	1.16/1.59	NS

*Note*: Quantities of immunoglobulins are expressed as medians in g/L.

Abbreviation: NS, not significant.

**TABLE 5:** Comparison of patients after treatment with patients with stable disease.

Immunoglobulin class	Stable	After treatment	*p* value
IgG	9.86	6.61	0.0005
IgA	1.53	0.74	0.0001
IgM	0.57	0.32	0.0021
IgG1	5.63	4.13	0.0026
IgG2	2.85	1.97	0.0008
IgG3	0.43	0.38	NS
IgG4	0.31	0.16	0.0018
IgA1	1.3	0.58	0.0005
IgA2	0.3	0.19	0.0025

*Note*: Quantities of immunoglobulins are expressed as medians in g/L.

Abbreviation: NS, not significant.

We also separately analyzed the paired samples of patients who were treated with chlorambucil‐based regimens (*n* = 20; 11 × R‐Clb, 9 × O‐Clb), BR (*n* = 17) and FCR (*n* = 18). Only in the FCR group did treatment result in significant rise of IgA (median 0.52 g/L vs. 0.58 g/L, *p* = 0.018), IgG2 (median 1.67 g/L vs. 1.78 g/L, *p* = 0.047), and IgA1 (median 0.44 g/L vs. 0.49 g/L, *p* = 0.048). Changes after BR and chlorambucil‐based regimens were not statistically significant.

### 3.2 Relation to infections and prognostic significance

In the 3‐year period prior to blood collection, infection of any grade occurred in 22/45 stable patients. Sites of infection are described in Table 6. Ig levels of patients with and without infections were not significantly different (data not shown).

**TABLE 6:** Frequency of different infectious episodes.

Site of infection	Number of episodes
Stable patients	
Upper respiratory tract infection and bronchitis	12
Herpes labialis	3
Urinary tract infection	2
Pneumonia; sepsis; infectious endocarditis; erysipelas; herpes zoster	1
Patients with progressive disease	
Upper respiratory tract and bronchitis	21
Pneumonia	10
Herpes labialis	4
Phlegmon	3
Herpes zoster; fever of unknown origin	2
Urinary tract infection; peritonsillar abscess; gastroenteritis; oropharyngeal candidiasis; clostridial colitis; cytomegalovirus stomatitis	1

Out of 87 patient with progressive disease, 29 had experienced infections within the 3‐year period prior to blood collection. As nine patients had multiple infections, there were a total of 48 episodes of infections (see Table 6 for details). Again, differences between Ig levels of patients with and without infections did not reach statistical significance.

As grade 3 or higher infections occurred only in four patients with stable disease and six patients with progressive disease, they were not analyzed separately.

Progression to treatment occurred in 12/45 stable patients and three of them died. The TTFT at 3 years was 79%. None of the Ig levels were significantly associated with TTFT, although there was a borderline *p* value in the case of IgA2 (*p* = 0.056).

**FIGURE 3:**
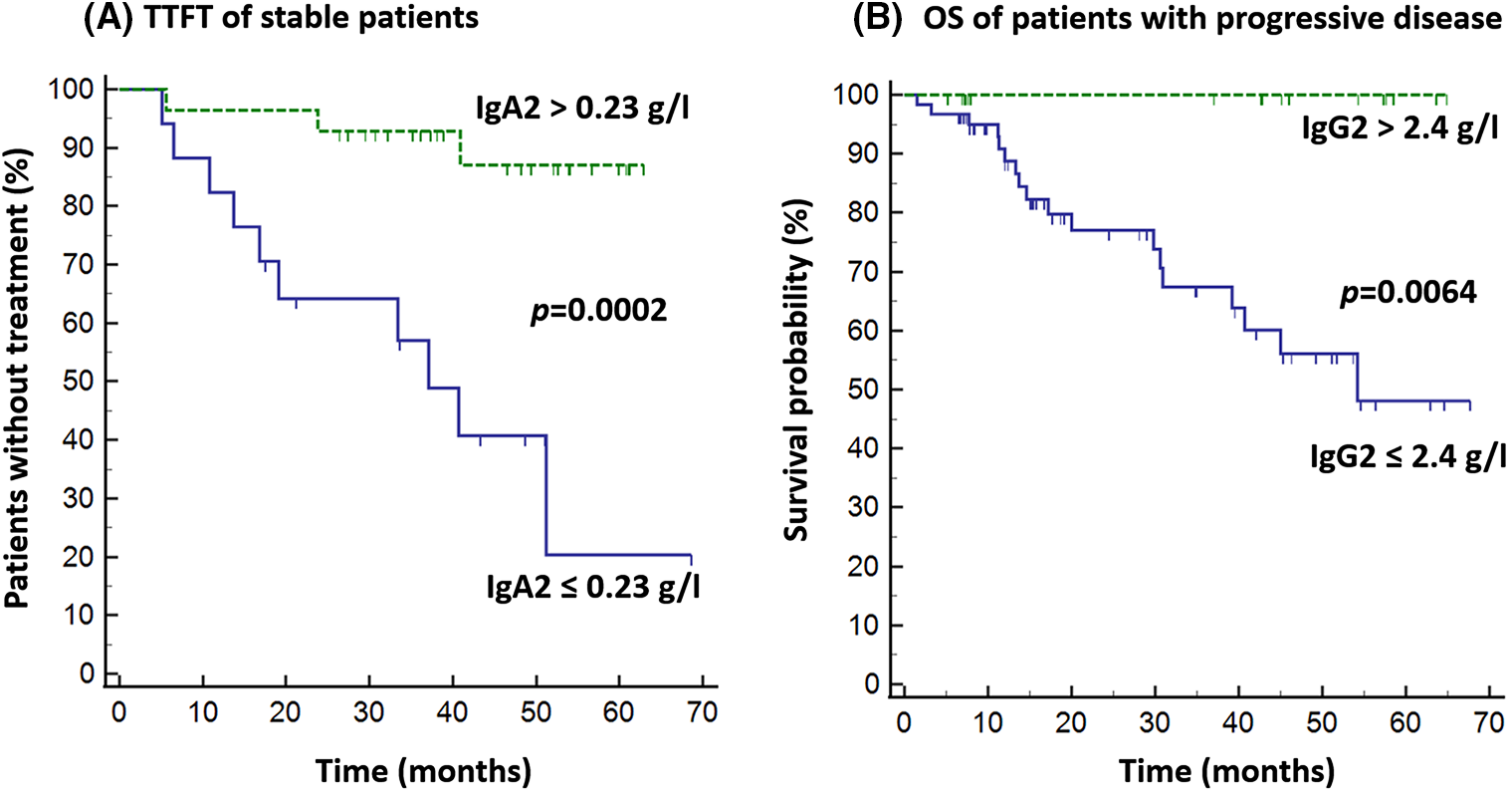
(A) Differences between TTFT of stable patients based on IgA2 level. ROC analysis set a cut‐off level for the best separation of curves as 0.23 g/L. In the log‐rank test, patients with IgA2 ≤ 0.23 g/L had a shorter TTFT (*p* = 0.0002). (B) Differences between OS of patients with progressive disease based on IgG2 level. ROC analysis set a cut‐off level for the best separation of curves as 2.4 g/L. In the log‐rank test, patients with IgG2 ≤ 2.4 g/L had shorter OS (*p* = 0.0064).

Within the cohort of progressive CLL, 18/87 patients died and three‐years OS was 78%. Shorter overall survival was observed in patients with lower IgG2 (*p* = 0.043). We performed ROC analysis to separate stable patients based on IgA2 level and patients with progressive disease based on IgG2 level into two groups with different TTFT and OS (Figure 3). We did not observe any association between Ig levels and TTNT or OS in 55 patients treated with CIT.

Prognostic significance of increase in Ig quantity after treatment was also investigated. The increase was expressed as fold‐change caused by treatment. In this analysis, higher increase of IgG2 was associated with shorter OS (*p* = 0.033). Other classes or subclasses of Ig had no influence on neither OS nor TTNT of treated patients.

### 3.3 Association with prognostic markers

For patients with stable disease, the Ig levels of those with mutated and unmutated IGHV did not differ significantly. Other unfavorable prognostic factors (17p deletion, *TP53* mutation and advanced Rai stage) were rarely present among stable patients – only two of them had advanced Rai stage, and none had 17p deletion or *TP53* mutation. ALC inversely correlated with levels of IgA (*p* = 0.014) and IgM (*p* = 0.0024). There was no association between Ig levels and sex or age of stable patients.

Among patients with progressive disease, those with mutated IGHV had lower levels of IgG (median 5.09 vs. 7.39 g/L; *p* = 0.0049), IgM (median 0.16 vs. 0.47 g/L; *p* = 0.0006), and IgG1 (median 3.38 vs. 4.78 g/L; *p* = 0.012). Patients with progressive disease and *TP53* mutation or 17p deletion had higher levels of IgA (median 0.87 vs. 0.56 g/L; *p* = 0.0093) and IgA1 (median 0.81 vs. 0.47 g/L; *p* = 0.01). No difference in Ig levels was observed among patients with progressive disease based on sex or Rai stage (stage 0–II vs. stage III–IV). ALC inversely correlated with levels of IgA (*p* = 0.033), IgG2 (*p* = 0.0074), IgA1 (*p* = 0.048) and IgA2 (*p* = 0.0031). There was also inverse correlation between age and IgG2 levels (*p* = 0.034).

When fold‐changes after treatment were compared among groups of treated patients divided based on prognostic factors, only significant result was higher increase of IgA2 in patients with advanced disease (0.86‐fold decrease in patients with Rai stage 0–II vs. 1.67‐fold increase in patients with Rai stage III–IV, *p* = 0.0071).

## 4 DISCUSSION

CLL and its treatment are associated with complex alteration of the immune system with a possible impact on the disease course, the risk of infections and thus the patients’ prognosis. We present an analysis of Ig quantities, including subclasses, in patients with CLL before and after the first‐line treatment with CIT.

In different studies, the prevalence of hypogammaglobulinemia in untreated patients was described, ranging from 9.9% to 34.7% for IgG, 12%–29.5% for IgA and 14.8%–54% for IgM.^6^, ^7^, ^8^, ^9^, ^10^, ^11^, ^12^, ^13^, ^14^ Our results in patients with stable disease fit into this range, while in patients with progressive CLL, we found a higher prevalence of hypogammaglobulinemia for IgG (55%) and IgA (63%). Most of the published data are on patients at the time of diagnosis, which could explain the difference from our progressive cohort. In studies by Mauro et al. and Parikh et al., the prevalence of IgG deficiency increased during follow‐up.^6^, ^7^ We did not repeat measurements during follow‐up (except for measurement right after treatment) but compared these with stable CLL with those indicated for treatment. The latter had significantly lower quantities of all Ig classes and subclasses. This might not be surprising, but as far as we know, a similar comparison has not yet been published.

Lower IgG has been associated with shorter OS in studies by Rozman, Andersen and Crassini.^9^, ^10^, ^15^ Many other authors did not confirm this.^6^, ^7^, ^8^, ^11^, ^12^, ^16^, ^17^ More often, an association between shorter OS and low levels of Ig was found for the IgA class.^9^, ^10^, ^11^, ^12^ However, even this was not confirmed in all studies.^6^, ^8^, ^17^ Only Andersen et al. observed shorter OS in patients with IgM deficiency, while others did not.^6^, ^8^, ^9^, ^10^, ^11^, ^12^, ^15^, ^17^ Some studies evaluated the association of low Ig with shorter treatment‐free survival or TTFT. Parikh et al. found such an association for IgG (but they did not investigate other classes), Andersen et al. found one for IgM, and most others found one for IgA.^6^, ^7^, ^10^, ^11^, ^12^ In the publications of Singh et al. and Francis et al., no such association was found.^8^, ^17^ In our cohort, we observed a trend towards shorter TTFT in stable patients with lower IgA2 and an association between low IgG2 and shorter OS in patients with progressive disease (Figure 3). Treated patients with higher relative increase in IgG2 also had shorter OS, which probably only reflects their lower pretreatment levels of this subclass. None of the main Ig classes had any prognostic value in our study, but it has to be said our number of patients was much lower than that in some abovementioned publications. Based on these studies, the association between Ig levels and prognosis remains controversial, but most evidence highlights the significance of IgA values.

Many of the aforementioned studies investigated the possible association of low Ig and infections, and the results were similarly ambiguous. Some studies did not find such an association in untreated patients with hypogammaglobulinemia.^10^, ^13^, ^18^ However, Andersen et al., with thus far probably the largest investigated cohort of 1204 untreated patients, observed a higher risk of infections in patients with low IgG, IgA, and IgM.^14^ Low IgA remained a significant risk factor even in multivariate analysis. In a study by Visentin et al., a higher frequency of major infections (requiring intravenous antibiotics or hospitalization) was associated with a combined deficiency of IgG with IgA or IgM.^19^ Additionally, Francis et al. and Freeman et al. observed more infections in patients with hypogammaglobulinemia, although some of them were pretreated, unlike in other works.^17^, ^20^ In a cohort of 899 patients in Binet A stage CLL, the levels of Ig at the time of diagnosis had no association with infection; however, infections were more common in patients who developed hypogammaglobulinemia during follow‐up (only IgG was assessed in this way).^6^ Finally, Ishdorj et al., who assessed a need for Ig replacement therapy rather than the frequency of infections, identified low IgG and IgA as independent predictors for future Ig replacement.^12^ We did not find any association between Ig levels and the frequency of infections, but again, the number of patients in our analysis was quite low, and more studies are necessary to definitively resolve this matter. The connection between Ig deficiency and infections might be difficult to establish because hypogammaglobulinemia is certainly not their only cause, and the inability of B cells to form a specific response to new antigens may also play a role.^43^ This is in line with the known lack of efficacy of vaccination in CLL.^44^, ^45^ Naturally, numerous other changes in the immune system that occur during the disease course contribute to infections as well.^25^

We evaluated the possible association of Ig levels and the most widely used prognostic factors—Rai stage, IGHV mutational status, *TP53* mutation, and deletion of 17p. Similar analyzes are quite rare in the literature. Parikh et al. observed lower IgG in patients in Rai stage III–IV, with higher CD49d expression and trisomy 12.^7^ Other cytogenetic aberrations, age, sex, ZAP70 and CD38 expression or IGHV mutational status had no association with IgG levels. Neither of these factors, including Rai stage and β2‐microglobulin, were associated with IgG levels in a study by Mauro et al., but low IgA was more frequently found in patients with splenomegaly and CD38 positivity and low IgM was found in those with higher lymphocyte counts and in Rai stage 0–II.^6^ Ishdorj et al. found IgG and IgA deficiency more often in advanced Rai stage and in patients with IGHV 1‐69, 3‐21, and 3‐49 subtypes.^12^ Low levels of IgA were also associated with CD38 positivity and elevated β2‐microglobulin, while abnormal IgM was associated with CD38 positivity and mutated IGHV. Cytogenetics were not assessed in this study. Finally, Singh et al. observed IgA deficiency in patients with advanced Rai stage and higher leukocyte count.^8^ Again, chromosomal aberrations were not assessed.

In our study, patients with CLL stage 0–II had levels of Ig comparable to those with stage III–IV. Here, we differ from the findings of Parikh et al., Ishdorj et al. and Singh et al., but the explanation is simple. We divided patients into a stable cohort and a cohort with progressive disease. In the former, all but two patients were in the early disease stage, and this analysis was not performed. In the latter, no difference was found, but even patients with Rai stage I or II had some indication for treatment (e.g., massive lymphadenopathy) and therefore their disease activity was probably similar to that in patients with Rai stage III–IV. Indeed, when we evaluated all patients together, we observed lower levels of all Ig classes and subclasses, with the exception of IgG1 and IgG3, in patients with Rai stages III–IV (data not shown). Comparison of patients with stable and progressive disease proved to be a better discriminating factor, as all Ig subclasses, including IgG1 and IgG3, were significantly different between the two groups.

A rather surprising finding from our analysis is that out of patients with progressive disease, those with unfavorable prognostic factors had higher levels of some classes or subclasses of Ig. Patients with unmutated IGHV had higher levels of IgG, IgG1 and IgM, and those with TP53 mutation or 17p deletion had higher levels of IgA and IgA1. One possible explanation is that hypogammaglobulinemia is not directly connected to aggressiveness of the disease but rather to its duration. CLL with unfavorable biological features usually progresses more rapidly, and some changes to the immune system, including those resulting in hypogammaglobulinemia, might actually need more time to fully develop. To the best of our knowledge, our study is the only one that examined the possible influence of *TP53* dysfunction. These results warrant further research on larger patient groups.

The most remarkable finding in our study is an increase in IgA levels after CIT administration. For the whole cohort of CIT‐treated patients, median IgA increased from 0.59 g/L to 0.74 g/L (*p* = 0.0031), while other Ig levels did not change significantly. When we assessed treatment regimens separately, only in the FCR group did we observe an increase in IgA, IgG2 and IgA1, while changes after BR and chlorambucil‐based regimens did not reach statistical significance. This may indicate that IgA increase in a whole cohort is mostly because of FCR‐treated patients. Nevertheless, the numbers of patients in subgroups with different regimens were very small. Higher relative increase of IgA2 after treatment in patients with Rai stage III–IV compared to patients with stage 0–II (where there was actually decrease) is surprising, as base values before treatment between the groups were similar. The reason is not clear; however, as it was observed only in one minor subclass out of all the Ig subclasses, the importance of this finding is probably limited.

IgA increase in CLL patients was repeatedly described after treatment with ibrutinib.^34^, ^35^, ^36^, ^37^, ^38^ In some of these studies, patients with an increase in IgA also had fewer infections.^36^, ^38^ In a randomized trial of ibrutinib versus ibrutinib plus rituximab, an increase in IgA was observed in both arms.^46^ Interestingly, IgG decreased in both arms, and IgM decreased in the combination arm (in other cited studies, IgG and IgM remained unchanged after ibrutinib). In contrast to BTKi, most studies report a decrease in Ig levels after CIT. Data from the era before the introduction of anti‐CD20 treatment are fairly limited. In an analysis that included three different studies using fludarabine (*n* = 71) or fludarabine with prednisone (*n* = 103) as first‐line treatment of CLL, the majority of patients had significant increases in their IgG and IgM levels.^27^ IgA levels mostly remained stable, although an increase was still more common than a decrease. These changes were independent of treatment response. More recent reports mostly encompass data from studies adding anti‐CD20 treatment to chemotherapy. Keating et al. monitored Ig levels in their trial of FCR for treatment‐naïve patients with CLL.^28^ They had no statistically significant change in IgG, IgA or IgM levels 6 months after treatment initiation. At 12 months, only IgG significantly decreased compared to the level before treatment (all three measurements of IgG level were performed in 105 patients). There was no association with treatment response. As the number of reports on Ig levels after CLL treatment is limited, we also included data from studies with other diagnoses. In a retrospective analysis of 211 patients with lymphomas (only 38 were CLL/small lymphocytic lymphoma) treated with a combination of rituximab and chemotherapy, the percentage of patients with low IgG increased from 15% to 48% after treatment.^29^ The proportion of patients with low IgA increased from 11% to 33% and that of patients with low IgM increased from 24% to 52%. A higher incidence of hypogammaglobulinemia was associated with a higher dose of rituximab and with fludarabine‐containing chemotherapy but not with age, sex, or lymphoma type. In a small series of patients with indolent lymphomas treated with fludarabine and rituximab, 24 of 27 patients had no significant change in their IgG, IgA, or IgM levels.^30^ A large analysis of the effect of rituximab on IgG levels was performed on 243 patients with autoimmune conditions, mostly vasculitis.^31^ More than half of them also received oral cyclophosphamide. Before rituximab administration, 26% of patients had IgG <7 g/L, which increased to 56% during follow‐up. There was no association between the total dose of rituximab and IgG nadir. The total dose of cyclophosphamide was associated with the IgG nadir in period after rituximab administration but not before it.^31^ Data on bendamustine or obinutuzumab treatment are scarce. In the GALLIUM study, patients with follicular lymphoma had a decrease in all Ig classes regardless of the antibody used or chemotherapy (bendamustine or cyclophosphamide with doxorubicin, vincristine and prednisone), but exact values were not published.^32^ In the GAUDI study, patients with follicular lymphoma received the same chemotherapy regimens combined with obinutuzumab only.^33^ The median levels of all Ig classes decreased after the induction phase of the treatment but remained within the normal range and relatively steady during obinutuzumab maintenance. No information is given on possible variation between different chemotherapies used. Taken together, apart from the trial of Keating et al., we do not have any data on Ig levels in large cohorts of CLL patients treated with currently used CIT regimens.

A possible explanation for the IgA increase after CIT administration lies in the general reconstitution of immune functions after suppressing the CLL clone. Even after ibrutinib treatment, where it has been described repeatedly, this phenomenon is poorly understood. Suggested mechanisms involve increased availability of growth factors for nonclonal B cells and their resulting increase after CLL cell elimination, changes in the T cell repertoire, or a shift from a Th2 to Th1 immune response.^36^, ^47^, ^48^ Although the Th1 type of immune response is based on cytotoxic T cells rather than antibody production, this shift was proven to correlate with IgA increase in ibrutinib‐treated patients.^48^ It is possible that some of these changes are not exclusive to ibrutinib and its mechanism of action but may be attributed to any therapy that leads to disease remission. Apparently, more research is still needed to clarify the mechanism of IgA increase after ibrutinib and, as our study shows, possibly also after CIT.

## 5 CONCLUSIONS

We performed a complex assessment of serum Ig levels in CLL patients, including the changes after the first‐line treatment with CIT. To the best of our knowledge, our study is the first to report a comparison of Ig levels between patients with stable, indolent disease and disease indicated for treatment. All Ig levels were significantly lower in the latter group. We present hitherto unpublished observations of higher Ig levels in patients with *TP53* dysfunction, possibly as a consequence of the shorter duration of a disease with an aggressive course. Finally, we complement scarce data on the impact of CIT on Ig levels in CLL. It appears that an increase in IgA levels described after ibrutinib may not be an exclusive feature of BTKi treatment but may be induced by CIT as well. Such an increase could at least partially be a consequence of suppressed disease activity. An IgA increase seems even more important in light of all the earlier published data suggesting a possible prognostic impact of IgA levels and their association with infectious complications. The major limitations of our study include the relatively low number of patients and its unicentric design. Thus, further studies in larger patient cohorts are warranted.

## AUTHOR CONTRIBUTIONS

**Pavel Vodárek:** Conceptualization (lead); investigation (lead); methodology (lead); project administration (lead); writing – original draft (lead). **Dominika Écsiová:** Investigation (supporting); methodology (supporting); writing – review and editing (supporting). **Vladimíra Řezáčová:** Formal analysis (supporting); investigation (supporting); methodology (supporting); writing – review and editing (supporting). **Ondřej Souček:** Formal analysis (supporting); investigation (supporting); methodology (supporting); writing – review and editing (supporting). **Martin Šimkovič:** Investigation (supporting); methodology (supporting); writing – review and editing (supporting). **Doris Vokurková:** Formal analysis (supporting); investigation (supporting); methodology (supporting); writing – review and editing (supporting). **David Belada:** Investigation (supporting); methodology (supporting); supervision (supporting); writing – review and editing (supporting). **Pavel Žák:** Investigation (supporting); methodology (supporting); supervision (supporting); writing – review and editing (supporting). **Lukáš Smolej:** Conceptualization (supporting); funding acquisition (lead); investigation (supporting); methodology (supporting); project administration (supporting); supervision (lead); writing – review and editing (lead).

## FUNDING INFORMATION

This work was supported by the Ministry of Health, Czech Republic under grant MH CZ‐DRO (UHHK, 00179906), by Charles University in Prague under grant Cooperatio, research area ONCO and by the League against Cancer Prague (unspecified grant).

## CONFLICT OF INTEREST STATEMENT

Pavel Vodárek reports consultations for Roche, Gilead, Janssen‐Cilag and Servier, research funding from Roche, honoraria and travel grants from AbbVie, Roche, Gilead, Janssen‐Cilag, Servier and Celgene. Martin Šimkovič reports consultancy fees, advisory board participation fees, travel grants, and honoraria from Janssen‐Cilag, Gilead, Roche, AstraZeneca, and AbbVie. David Belada reports consultancy fees, advisory board participation fees, travel grants and honoraria from Roche, Takeda, Gilead and Janssen‐Cilag. Lukáš Smolej reports honoraria, consultancy fees and travel grants from Roche, AbbVie, Janssen, and AstraZeneca. Other authors report no conflicts of interest.

## ETHICS STATEMENT

All patients signed the necessary informed consent forms, and the study was approved by the local ethics committee and conducted according to the principles of the Declaration of Helsinki.

## Supporting information


Table S1.

## Data Availability

The data that support the findings of this study are available on request from the corresponding author. The data are not publicly available due to privacy or ethical restrictions.
