# Supplementary material for: Complex evaluation of serum immunoglobulin levels in patients with chronic lymphocytic leukemia: Significant increase in IgA after first‐line chemoimmunotherapy
Source: Cancer Med. 2024 Aug 9;13(15):e7399. doi: 10.1002/cam4.7399 (PMC11310768; doi:10.1002/cam4.7399)
Supplement: Supplementary file 1 — Table S1. [file CAM4-13-e7399-s001.docx]

**Supplementary Appendix**

Supplementary Table 1. Comparison of patients with stable disease with controls. Quantities of immunoglobulins are expressed as medians in g/l. NS – not significant.

| **Immunoglobulin class** | Stable | Controls | *p* value |
| --- | --- | --- | --- |
| IgG | 9.86 | 11.5 | **0.026** |
| IgA | 1.53 | 2.3 | **<0.0001** |
| IgM | 0.57 | 0.84 | **0.0044** |
| IgG1 | 5.63 | 6.88 | **0.016** |
| IgG2 | 2.85 | 3.8 | **0.026** |
| IgG3 | 0.43 | 0.34 | NS |
| IgG4 | 0.31 | 0.55 | **0.0045** |
| IgA1 | 1.3 | 2.11 | **0.0003** |
| IgA2 | 0.3 | 0.75 | **0.0033** |

Supplementary Table 2. Comparison of patients with progressive disease with controls. Quantities of immunoglobulins are expressed as medians in g/l. NS – not significant.

| **Immunoglobulin class** | Progressive | Controls | *p* value |
| --- | --- | --- | --- |
| IgG | 6.96 | 11.5 | **<0.0001** |
| IgA | 0.63 | 2.3 | **<0.0001** |
| IgM | 0.36 | 0.84 | **<0.0001** |
| IgG1 | 4.22 | 6.88 | **<0.0001** |
| IgG2 | 1.76 | 3.8 | **<0.0001** |
| IgG3 | 0.28 | 0.34 | NS |
| IgG4 | 0.17 | 0.55 | **<0.0001** |
| IgA1 | 0.55 | 2.11 | **<0.0001** |
| IgA2 | 0.18 | 0.75 | **<0.0001** |

Supplementary Table 3. Comparison of patients after treatment with controls. Quantities of immunoglobulins are expressed as medians in g/l. NS – not significant.

| **Immunoglobulin class** | After treatment | Controls | *p* value |
| --- | --- | --- | --- |
| IgG | 6.83 | 11.5 | **<0.0001** |
| IgA | 0.74 | 2.3 | **<0.0001** |
| IgM | 0.36 | 0.84 | **<0.0001** |
| IgG1 | 4.2 | 6.88 | **<0.0001** |
| IgG2 | 1.97 | 3.8 | **<0.0001** |
| IgG3 | 0.39 | 0.34 | NS |
| IgG4 | 0.16 | 0.55 | **<0.0001** |
| IgA1 | 0.65 | 2.11 | **<0.0001** |
| IgA2 | 0.19 | 0.75 | **<0.0001** |
